# Supplementary material for: DMRforPairs: identifying Differentially Methylated Regions between unique samples using array based methylation profiles
Source: BMC Bioinformatics. 2014 May 15;15:141. doi: 10.1186/1471-2105-15-141 (PMC4046028; doi:10.1186/1471-2105-15-141)
Supplement: Additional file 2 — DMRforPairs output for the comparison of A431-MCF7 and NA17018-NA17105. Please start from the HTML files in each folder. Available via the BMC Bioinformatics website. [file 1471-2105-15-141-S2.zip › 1394847754114233_MOESM2_ESM/A431_MCF7/figures/19006.pdf]

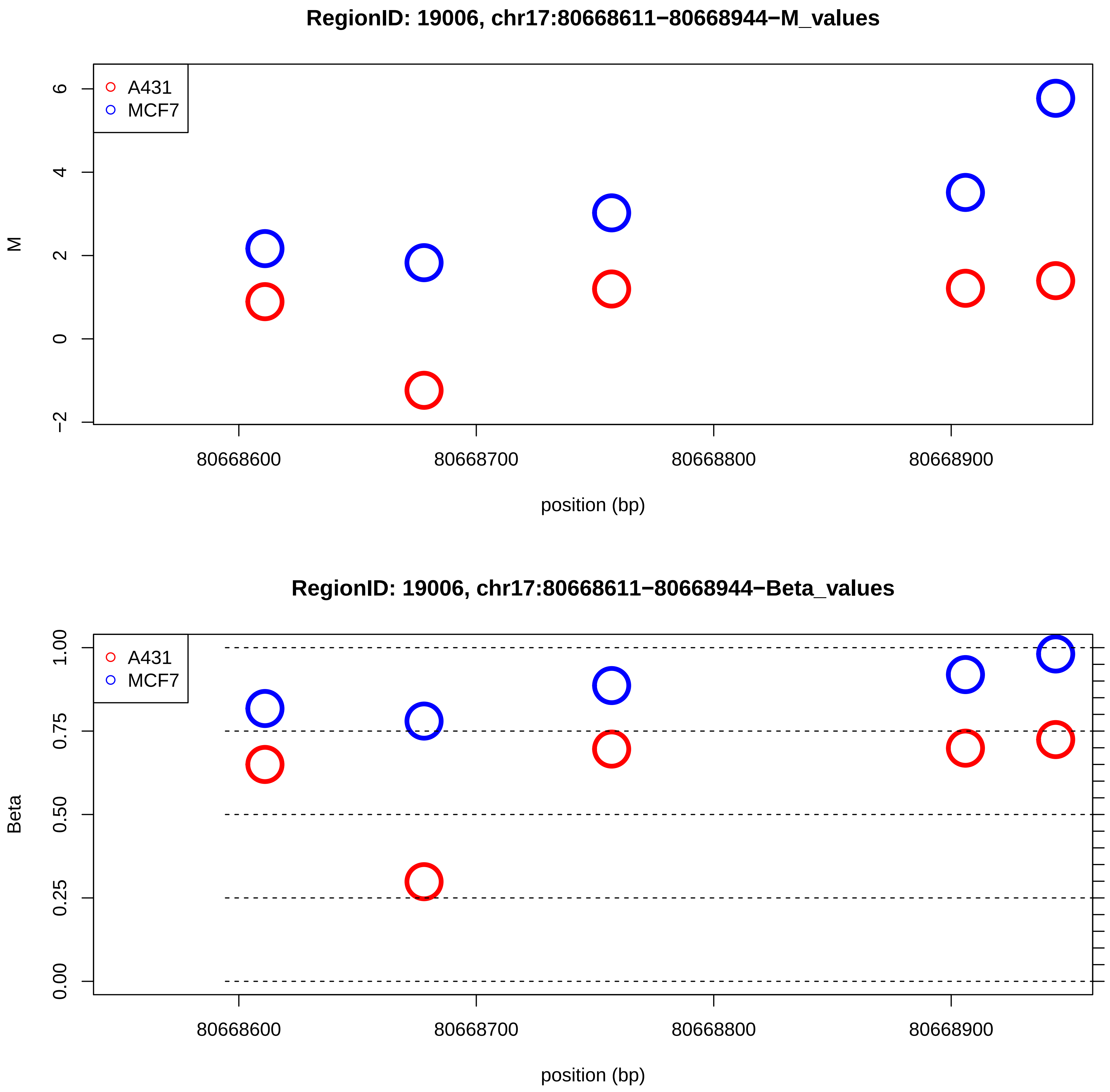
RegionID: 19006, chr17:80668611−80668944−M_values
A431
MCF7
M
−2
0
2
4
6
80668600
80668700
80668800
80668900
position (bp)
RegionID: 19006, chr17:80668611−80668944−Beta_values
A431
MCF7
Beta
0.00
0.25
0.50
0.75
1.00
80668600
80668700
80668800
80668900
position (bp)

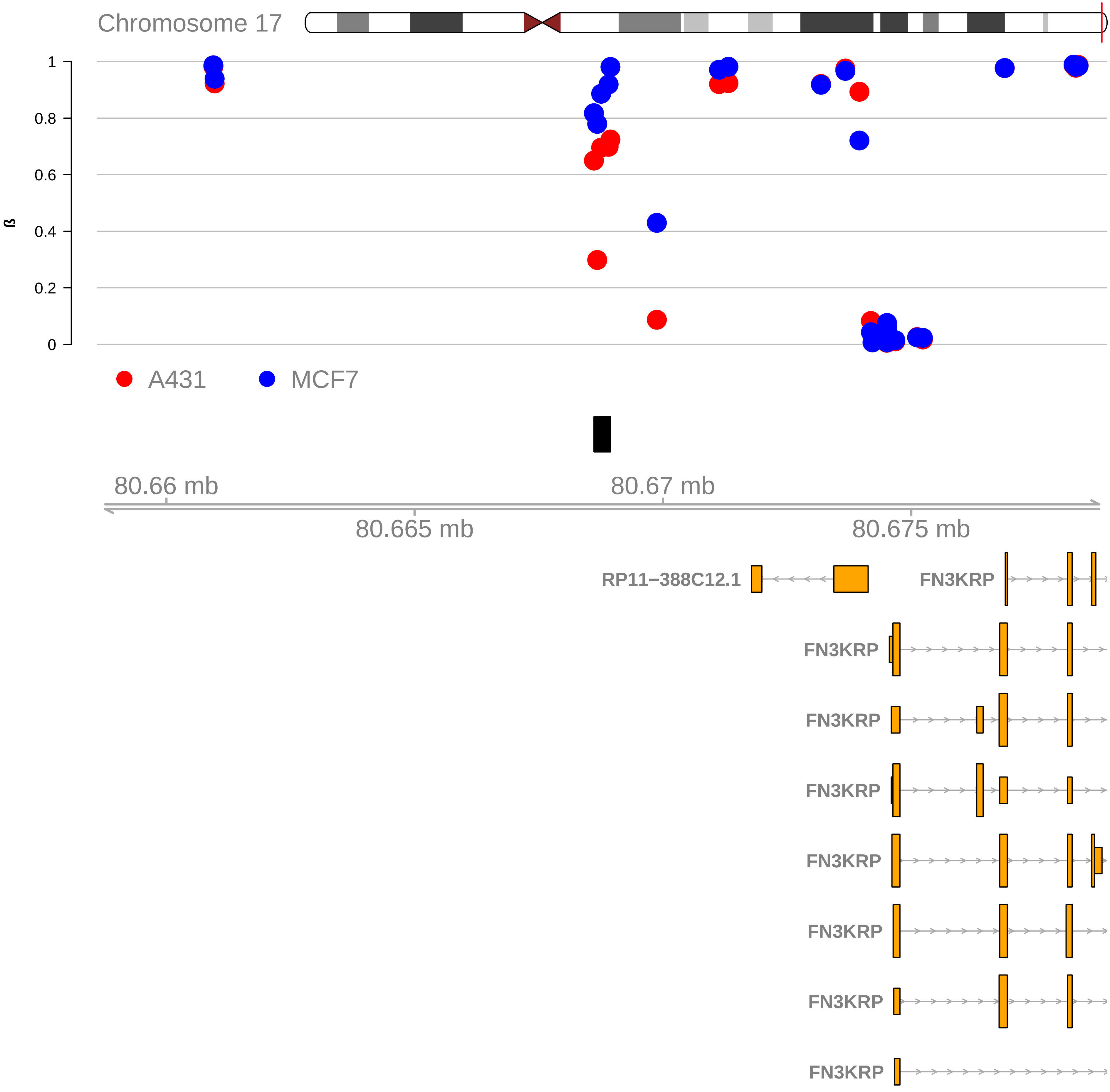

Chromosome 17
1
0.8
0.6
0.4
0.2
0
ß
A431
MCF7
80.66 mb
80.67 mb
80.665 mb
80.675 mb
RP11−388C12.1
FN3KRP
FN3KRP
FN3KRP
FN3KRP
FN3KRP
FN3KRP
FN3KRP
FN3KRP
